# Intersectionality in informal care research: a scoping review

**DOI:** 10.1177/14034948211027816

**Published:** 2021-07-07

**Authors:** Aldiene H. Hengelaar, Yvette Wittenberg, Rick Kwekkeboom, Margo Van Hartingsveldt, Petra Verdonk

**Affiliations:** 1 Department of Ethics, Law and Humanities, Amsterdam Public Health Research Institute, The Netherlands; 2 Department of Occupational Therapy, Amsterdam University of Applied Sciences, The Netherlands; 3 Faculty of Social and Behavioral Sciences, University of Amsterdam, The Netherlands; 4 Faculty of Applied Social Sciences and Law, Amsterdam University of Applied Sciences, The Netherlands; 5 Department of Occupational Therapy, Amsterdam University of Applied Sciences, The Netherlands

**Keywords:** intersectionality, informal care, diversity, care networks, collaboration

## Abstract

*Aim:* Informal caregivers share common experiences in providing care to someone with health and/or social needs, but at the same time their experiences differ across diverse backgrounds such as gender, age, culture, as these aspects of diversity co-shape these experiences. This scoping review aims to explore how aspects of diversity, across their intersections, are currently incorporated in informal care research and discusses how an intersectional perspective can further develop our understanding of informal care. *Methods:* A scoping review was performed to map relevant caregiving literature from an intersectionality perspective. Key terms ‘informal care’ and ‘intersectionality’ were used for a search in four databases resulting in the inclusion of 28 articles. All 28 studies were analysed based on a scoping review created intersectionality informed coding scheme. *Results:* Aspects of diversity are largely understudied in informal care research, in particular across their intersections and from a critical perspective. This intersectional informed analysis revealed that when studying diverse caregiving experiences the use of intersections of dimensions of diversity provides a nuanced understanding of these experiences. *Conclusions:* **Adopting an intersectional perspective ensures that not only different categories or social identities of caregivers are included in future studies, but the mutual relationships between these categories embedded in their specific context are actually studied.**

## Background

Informal care responsibilities have increased in the past decades [1] based on policy and societal developments, such as ageing societies, deinstitutionalisation and changes in the family and working life of citizens [2–4]. Informal caregivers share common experiences in providing care to someone with health and/or social needs and simultaneously their experiences are unique. The experiences of caregivers are lived and therefore subject to diversity. Diversity generally refers to a plurality of categories and focuses on structural group memberships that people belong to [5]. Within the field of informal care research, studies show that multiple dimensions of diversity such as age, gender, religion, marital or household status, education level, socioeconomic status and ethnicity influence caregiving [6–10]. Diversity aspects play a role in underlying expectations, values and norms, assumptions and behaviours regarding the provided care within collaborative care networks [8, 9]. For example, adequately understanding differences in caregivers’ experiences and assumptions may prevent possible negative consequences of contradicting expectations between informal and formal care providers, improving good collaboration between them [9]. This stresses the importance of paying attention to differences in roles, expectations and needs of all actors in care networks. As good collaboration in care networks increases the chance of good quality care, the perceived wellbeing of care recipients and caregivers and reduces caregiver burden [11].

Categories of diversity are socially constructed and power laden [12]. For example, the role of gender is widely described in caregiving research showing gender hierarchies exist which entail that more status and power is assigned to what is considered more masculine. Hence, gender must be understood not as a characteristic of the individual, but rather as a relation between groups. Furthermore, gender takes place at multiple levels, such as at the intra-psychological level conceived of for instance internalised norms and behaviours. Gender can also take place at the institutional level, where it structures how caregiving is organised and who takes care of whom, how, when, and whether caregiving is paid for or not [13]. This example of gender shows that the provision of informal care takes place in a broader context, and can be understood as a type of relationship between individuals which is embedded within socially and politically defined sets of expectations and practices regarding rights and responsibilities [6, 14]. Other studies show, for example, the role of culture, or age, or geography in relation to informal caregiving [6, 8]. This shows that a wide range of diverse experiences can be found among caregivers, associated with group memberships. However, people do not live single-issue lives. Caregivers are a heterogenous group which consists of individuals who are members of multiple social groups. They are women or men of certain ages and with particular cultural backgrounds and education levels, creating unique social positions that inform caregiving experiences [6].

Intersectionality, originally coined by Crenshaw in 1989, refers to the interactions between dimensions of diversity in individual lives, social practices, institutional arrangements, and cultural ideologies and the outcomes of these interactions in terms of power and social inequities [15]. Intersectionality is a major theoretical and research paradigm across a multitude of disciplines including health and healthcare research [16]. Although the additional value of intersectionality seems evident in both qualitative and quantitative research to understand caregiving better [6, 11, 17, 19], it is understudied in caregiving research [6, 18]. This requires to look beyond single categories of diversity and analyse how dimensions of diversity interact with each other to shape caregivers’ experiences. Such an approach provides more opportunity to analyse the complexity of caregiving and contributes to the provision of more tailor-made support for informal caregivers rather than generalised solutions [20, 21]. By looking at how caregiving issues are shaped by the interaction of different dimensions of diversity as well as by situational and contextual dimensions [6, 11, 22], ‘practices that privilege any specific axis of inequality’ [17, p. 1712] within informal care can be challenged.

This scoping review explores how dimensions of diversity across their intersections are currently represented in informal care research.

## Methods

A scoping review was conducted based on the framework developed by Arksey and O’Malley [23] to map relevant caregiving literature using intersectionality. The framework’s stages were followed: (1) identifying the research question; (2) identifying relevant studies; (3) study selection; (4) charting the data; (5) collating, summarising and reporting the results; and (6) consultation of stakeholders, such as key informants, care recipients, caregivers and practitioners [23]. The sixth stage is additional and not within the scope of this review. Stakeholder consultations will be performed at a later stage as this review is part of a larger research project of the first two authors.

In Arksey and O’Malley’s stage 2, key concepts of the search strategy were identified. First, ‘informal care’ and ‘intersectionality’ were identified as key concepts, synonyms were used for informal care, such as informal support, family care and caregiving. As it turned out, no studies within our scope explicitly mentioned the key concept intersectionality. Therefore, search terms were expanded by formulating a broad range of concepts that are often used as synonyms for this concept, including ‘diversity’ and ‘socioeconomic factors’, and by incorporating single dimensions of diversity (such as age, gender, and ethnicity). The final search strategy was used in four databases: PubMed, PsycINFO, Cochrane Library and CINAHL.

In stage 3, the databases were searched using the concepts and keywords identified in stage 2 and applying two inclusion criteria: (a) articles published in peer reviewed journals between January 2008 and March 2018; and (b) articles published in English or Dutch. Because of the large number of articles found, doubts about the relevance of some articles and to ensure a broad diversity lens in the included articles making it more likely that intersections would be analysed, it was decided post-hoc to use a third inclusion criterion, namely, (c) studies using a minimum of four dimensions of diversity. There was no pre-set demarcation of which dimension of diversity should be studied in the included studies. Table II shows the dimensions of diversity which were included in the studies. Studies in which informal care was provided in all kinds of contexts (community, residential, etc.) and to all types of care recipients were included. Empirical studies were not excluded based on research methodology.

**Figure 1.**
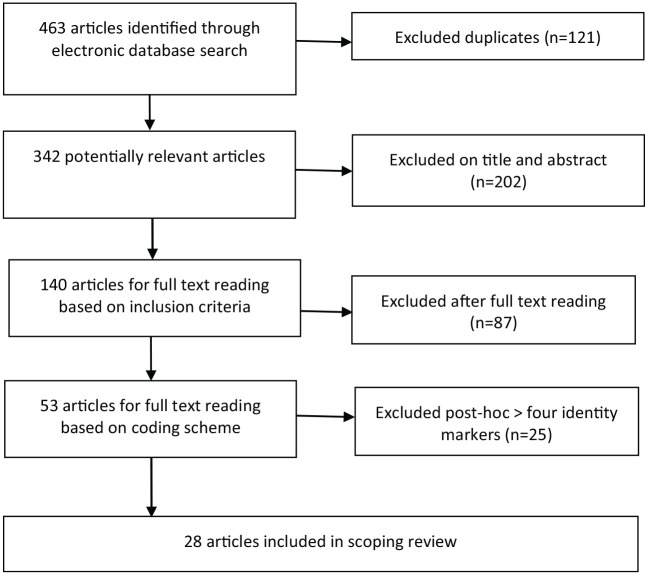
Preferred Reporting Items for Systematic Reviews and Meta-Analyses (PRISMA) flow chart.

All articles were selected based on title and abstract and subsequently based on their full text. This selection was performed independently by the first two authors and a junior researcher. This resulted in a collection of 53 articles. After applying the additional third inclusion criterion, 28 articles were selected to include in this scoping review. The Preferred Reporting Items for Systematic Reviews and Meta-Analyses (PRISMA) flow chart is presented in Figure 1.

For the next stage, charting the data, a coding scheme was created by the first two authors. The coding scheme is based on two primers on intersectionality informed research [16, 22] and ‘The complexity of intersectionality’ in which McCall (2005) describes three empirical analytical approaches to intersectional research [24]. Combining the key principles of conducting research from an intersectional perspective and the aim of our scoping review, the following was included in the coding scheme: (a) the consideration of intersectionality in the context in introductions, aims, samples and in the methods of data collection (including analytical approach); (b) used dimensions of diversity; and (c) the intersection of dimensions of diversity, thus investigating the intersectional identity matrix and the relatedness of diversity to the main conclusions of the included studies. The topics we inserted in our coding scheme are clarified in Table I.

**Table I.** Intersectionality informed coding scheme.

No.	Code	Description
1.	Context in introduction	Does the introduction consider the historical, cultural and societal/political context in line with the aim of the research? Does it consider the different power relations within and between the axes of diversity?
2.	Aim	Does the research question/aim consider an intersectional perspective? If not; what is the direction of the research question/aim?
3.	Sample	Sample size? Does the sample represent caregivers, care recipients, other actors in the social network and/or (healthcare) professionals? Does the sampling method reflect the complexity of social life? Have different dimensions of diversity been considered prior to the data collection?
4.	Method of data collection	How were the respondents approached? Is there mentioning of reflexivity of the researcher during the data collection? Method of data collection? Was an intersectional perspective considered while formulating the method of data collection?
5.	Identity markers	Which identity markers of caregivers are measured/considered? Is there also mention of contextual dimensions, care characteristics and identity markers of the care recipients? Are there arguments for the chosen intersections investigated?
6.	Analytical approach	Quantitative: Additive approach, multiplicative approach and interaction terms. Qualitative: Thematic synthesis, narrative, grounded theory, IPA, etc. Which analytical approach was used? e.g. based on McCall (2005) [3] anticategorical, intercategorical, intracategorical?
7.	Intersectional identity matrix	Do the identity axes intersect with each other in the analyses? How do these axes influence each other and the topic of research? Is attention given to the influence of power relations?
8.	Main conclusions	How is diversity related to the main conclusion of this study?

Based on Rouhani [16], Hunting [22] and McCall [24].

Following the Arksey and O’Malley framework, charted data were synthesised in order to present a narrative account of existing literature [23, 25]. Axial coding was applied based on the created coding scheme. Subsequently descriptive and overarching themes were developed, by using thematic synthesis, based on Thomas and Harden [26]. Performing a thematic synthesis while applying an intersectionality lens meant that the authors went beyond the themes of the primary studies [26]. Analysis was performed by the first and second author and a junior researcher (researcher triangulation). Disagreements about analysis were subject to discussion in the team of authors to reach consensus.

## Results

None of the 28 included articles mentioned intersectionality explicitly as a theoretical or analytical perspective. All studies included four or more dimensions of diversity when researching informal care, and 13 of the included studies researched intersections of dimensions of diversity. All study characteristics of the included studies are summarised in Table II.

**Table II.** Study characteristics of included studies.

Study	Country	Aim	In and exclusion criteria	Sample	Analytical approach	Method of data collection	Dimensions of diversity caregiver
Vroman & Morency (2011)	Belize	To develop a profile of the informal care of elders living with families. To identify and describe the characteristics of the informal caregivers, to achieve an understanding of their perceptions of caregiving and to recognise the socio-cultural factors that shaped their caregiving.	Not mentioned	*N*=29 informal caregivers of older adults in Belize	Thematic analysis	Semi-structured interviews	Age, gender, employment status, ethnicity, relationship, marital status, household situation, financial status
Ratcliffe, Lester, Couzner, & Crotty (2012)	Australia	Aims to utilise the ICECAP-O instrument to access the quality of life of a representative sample of the older South Australian population differentiated according to carer status, also determine the influence of several socio-demographic characteristics on the quality of life.	The socio-demographic variables included in the study were pre-selected by an external research organisation commissioned by the South Australian Department of Health. Sampling followed random selection of 5200 households. From each of the selected households, one person aged 15 years or older was randomly selected to participate.	*N*=786, caregivers *n*=115, non-caregivers *n*=671	Kruskal–Wallis test, Multivariate linear regression	Health Omnibus Survey, face-to-face survey	Age, gender, ethnicity, educational level, marital status, financial status, employment status, place of residence
Gupta, Isherwood, Jones, & van Impe (2015)	France, Germany, Spain, Italy, UK	This study examined health-related quality of life and comorbidities experienced by informal schizophrenia caregivers compared with non-caregivers and caregivers of adults with other conditions.	The NHWS is intended to represent the entire adult population of that country by employing a stratified random sampling framework. The demographic distributions of each country are obtained from the International Database from the United States Bureau of the Census; potential respondents are selected in such a way as to mirror these characteristics.	Total *n*=159.387, non-caregivers *n*=158.989, schizophrenia caregivers *n*=398	Chi-square, ANOVA, single logistic regression	National Health and Wellness Survey, annual, cross-sectional, self-administered questionnaire (2010, 2011 and 2013)	Age, gender, self-rated health, educational level, marital status, financial status, employment status, place of residence
Tokunaga & Hashimoto (2017)	Japan	The aim of this study was to examine the association between women’s SES and the likelihood of being a primary caregiver in Japan.	To focus on the within-gender gap, the male subjects were excluded from the analysis. The sample was limited to females aged between 40 and 60 years, because women in this age range are most likely to be involved in personal care but still be part of the labour force.	*N*=2399, female caregiver	Chi-square and t-test, multivariate logistic regression + interaction terms	Comprehensive Survey of Living Conditions of the People on Health and Welfare (2010 and 2013)	Age, gender, marital status, self-rated health, employment status, educational level, financial status, household situation
Vaingankar et al. (2016)	Singapore	This study aimed to describe the care participation, care needs, and care burden among informal caregivers and examine factors related to the care burden experienced by their informal caregivers.	Informants were excluded from the sample if (a) interviewers were unable to contact them after ten contact attempts, (b) they were non**-**residents of Singapore, residing outside Singapore at the time of the survey, (c) they were unwilling or unable to complete the interview in any of the four languages **–** English, Chinese (either Mandarin or one of three dialects **–** Hokkien, Teochew, or Cantonese), Malay, or Tamil, or (d) they were paid caregivers.	Total *n*=2421, *n*=693 informal caregivers	Mann-Whitney U test, X2-test, Multivariate logistic regression	Well-being of the Singapore Elderly survey (2013)	Age, gender, ethnicity, marital status, educational level, employment status, care arrangement, support of others, relationship, place of residence
Toribio-Diaz, Medrano-Martinez, Molto-Jorda & Beltran-Blasco (2012)	Spain	This article aims to describe the caregiver network providing service to dementia patients in the province of Alicante, Spain. It analyses characteristics of the patients receiving care in the network, as well as the profiles and roles corresponding to the different types of caregivers involved in patient care and management.	This study included families/caregivers of dementia patients monitored by these consults in consecutive order. Patients diagnosed a minimum of 1 year prior to recruitment, with any type of dementia at any stage, were included. We excluded patients diagnosed less than 1 year prior to recruitment; those whose PC or SC could not be contacted (they attended appointments alone or with a formal caregiver [FC]); patients residing in assisted-living centres; and patients whose relative/caregiver refused to sign the informed consent form.	Total *n*=303, *n*=129 primary caregivers, *n*=114 secondary caregivers, *n*=15 formal caregivers, *n*=45 absent caregivers	Chi-square test, t-test	Multi-centre prospective study in 4 general neurology departments	Age, gender, educational level, marital status, relationship, household situation, employment status, care motives, self-rated health
Eby et al. (2017)	United States of America	The goal of this research is to gain a better understanding of the characteristics of informal caregivers who provide transportation assistance and to explore the types and frequency of this assistance.	Samples were selected for the survey by first filtering the database for Michigan residents who were 45±80 years of age and randomly drawing replicate samples for the survey.	Informal caregivers *n*=268	T-test, Rao Scott modified chi square test	Telephone survey	Age, gender, marital status, household situation, ethnicity, financial status, educational level, employment status, self-rated health
Wang et al. (2016)	China	The aim of this exploratory study was to examine the prevalence and the related factors of depression among the female informal caregivers of disabled elders; to analyze the correlation of the depressive emotion between the informal caregivers’ care burden and disabled elders’ quality of life; and to investigate the influence of informal caregivers’ depressive emotion on informal caregivers’ care burden and the quality of life among disabled elders.	Informal caregivers were included if (1) they were 18 years or older; (2) they were the nominated family member of disabled elders aged 60 years and above; (3) they assisted in or supervised the ADL (i.e., personal care, mobility, communication, an emotional and/or practical and financial support) of the included elders; and (4) they provided unpaid family care. However, informal caregivers were excluded if they were cognitively impaired and incapable of completing the measurements. Moreover, disabled elders and their informal caregiver were excluded if either of them was unwilling to participate in this research.	Female Uyghur and Kazakh informal caregivers *n*=444	Multivariate logistic regression, Spearman’s RHO	Cross-sectional study: Self-Rating Depression Scale questionnaire, Social Support Rating Scale, Zarit burden interview, Katz ADL, SF-36 questionnaire, demographic characteristics self-report	Age, gender, ethnicity, marital status, educational level, relationship, employment status, financial status, household situation, place of residence, self-rated health
Sequeira (2013)	Portugal	The aim of this study is to determine the impact associated with caring for a dependent older person in terms of difficulties, coping strategies, sources of satisfaction and levels of burden and to compare the impact associated with caring for an older person with dementia versus a dependent older person without cognitive impairment.	Caregivers were selected from patients who used one geriatric psychiatry service and two general medical services. Both groups of caregivers were paired in terms of age, sex and level of dependency.	Informal caregivers *n*=184 (*n*=83 mental dependence, *n*=101 physical dependence)	Comparative analysis, linear regression, t-test	Questionnaire, Barthel Index, Lawton Index, Mini Mental State Examination, Clinical Dementia Rating, Caregiver Assessment of Difficulties Index, Caregiver Assessment of Managing Index, Caregiver Assessment of Satisfactions Index, Scale of Caregiver Burden	Age, gender, marital status, educational level, employment level, household situation
Hastrup, van den Berg & Gyrd-Hansen (2011)	The Netherlands	The aim of this study was to compare the subjective burden in a broad group of caregivers to care recipients with mental illnesses or a combination of mental and somatic illnesses, with caregivers to care recipients with somatic illnesses in order to test for a possible extra subjective caregiver burden in mental illnesses. The second aim was to test for an association between the subjective caregiver burden and caregivers’ characteristics and objective burden.	Not mentioned	Informal caregivers *n*=865	Chi-square test, t-test, interaction variables, multicollinearity	Postal questionnaire: Caregiver Strain Index, EQ-5D Index	Age, gender, educational level, employment status, financial status, relationship
Williams et al. (2008)	United States of America	The objectives of this study were to examine the relationship between selected decedent and caregiver characteristics, facility-related perceptions, and emotional and physical health of 434 informal of recently deceased residents of residential care/assisted living facilities and nursing homes. The potential mediating effects of social support (informal, staff, and spiritual) were also examined.	Not mentioned	Informal caregivers *n*=434	Linear mixed models	Telephone interviews	Age, gender, ethnicity, marital status, educational level, employment status, relationship
Tuithof, ten Have, van Dorsselaer,& de Graaf (2015)	The Netherlands	The aim of this study was to examine whether informal caregiving is associated with presence of any emotional disorder in the past year; and which characteristics (i.e. sociodemographic, caregiving-related and other characteristics) are risk indicators for any emotional disorder among informal caregivers.	Based on the most recent birthday at first contact within the household, an individual aged 18–64 years with sufficient fluency in the Dutch language was randomly selected.	General *n*=5.303, non-caregiver *n*=3.544, caregiver *n*=1.759	Logistic regression models, bivariate logistic regression	Netherlands Mental Health Survey and Incidence Study-2, face-to-face interviews	Age, gender, educational level, employment status, relationship, household situation
Schmidt et al. (2016)	Austria, Germany, Sweden, the Netherlands, Spain, Italy, France, Denmark, Switzerland, and Belgium	Focusing on care provision to grandchildren and (older) relatives (‘informal care’) as forms of engagement, this paper aims to identify which individual characteristics may compensate for health deficits and enable individuals with multimorbidity to provide informal care.	Informal caregiving is proxied by two binary dependent variables, indicating whether the individual has provided either of two types of informal care within the previous 12 months or during the time elapsed from the prior interview. The first refers to the provision of extra-residential care to adults. The second to grandchild care provision.	Extra-residential care *n*=13.020, Grandparent *n*=18.946	Multivariate logistic regression models	Survey of Health, Ageing and Retirement in Europe (2004-2005, 2006-2007, 2011-2012)	Age, gender, ethnicity, culture, educational level, employment status, household situation
Joyce, Berman, & Lau (2014)	United States of America.	The aim of this study was to explore factors related to caregivers’ support with managing medications for end-of-life home hospice patients.	Eligible caregivers had to (1) be unpaid, aged 18+ years, English-proficient, and self-identified as primary caregiver; (2) have medication responsibilities for a home hospice patient aged 60+ years; and (3) have no cognitive/sensory deficits precluding a telephone interview.	Informal caregivers *n*=120	Univariate analysis, bivariate generalised logistic regression	Computer-assisted telephone interviews	Age, gender, ethnicity, relationship, educational level, employment status, financial status, household situation
del Río-Lozano et al. (2013)	Spain	The aim of this study was to identify how gender roles and identity influence the assumption of the caregiving role in men and women and to analyze gender differences in dealing with caregiving and in health outcomes.	The study population was defined as primary informal (unpaid) live-in or live-out caregivers of a disabled person within their close social or family network. Only individuals were included who had been providing care for at least 6 m.	Informal caregivers *n*=32	Sociological discourse analysis, comparative analysis	Semi-structured interviews, selected people with different individual characteristics	Age, gender, educational level, employment status, place of residence, relationship
Kenny, Hall, & Davis (2011)	Australia	This article aims to advance the understanding of the health impacts of caregiving in the palliative care context. It reports a study that investigated associations between health and a range of caregiving context variables, which represent potential stressors or resources, among current informal carers of patients receiving palliative care at home.	Carers were eligible if they were English speaking, currently providing assistance to a patient receiving palliative care at home from one of the participating services, and the patient and carer provided consent.	Informal caregiver *n*=178	Ordinary least-squares regressions	A cross-sectional observational study of the HRQOL of the carers of patients receiving palliative care ( 2005–2006)	Age, gender, relationship, employment status, ethnicity
Verbakel et al. (2017)	Finland, Denmark, Norway, Sweden, France, Switzerland, Belgium, Netherlands, Germany, Czech Republic, Poland, Slovenia, Portugal, Estonia, UK, Spain, Ireland, Lithuania, Austria, Hungary.	The aim of this study is to offer a more complete picture of informal care, covering more types of relationships between care receiver and caregiver than the parent–child relationship and, consequently, also a more diverse set of reasons for providing informal care.	Not mentioned	Total *n*=28.406, Informal caregivers *n*=9.422	Logistic multilevel analysis, linear multilevel analysis	European Social Survey-7 collected through face-to-face interviews	Age, gender, marital status, employment status, educational level, household situation, religiosity, time spent caregiving
Doebler, Ryan, Shortall & Maguire (2017)	United Kingdom	The aim of this study is to address several gaps in the literature regarding the impact of caregiver burden, employment, gender, age and proximity to services on the relationship between informal care-giving and mental ill-health.	Not mentioned	Non-caregiver *n*= 321.972, caregiver *n*=56.393	Binary logistic multilevel models	Northern Ireland Longitudinal Study, Northern Ireland Enhanced Prescribing Database, Northern Ireland Statistics and Research Agency	Age, gender, marital status, educational level, employment status, place of residence, self-rated health
Robards, Vlachantoni, Evandrou & Falkingham (2015)	United Kingdom	The key aim of the study is to understand ‘what became of carers in 2001, 10 years later’.	Not mentioned	Non-caregiver *n*=240.979, caregiver *n*=76.953	Cross-sectional analysis	Office for National Statistics Longitudinal Study, (2001 &2011)	Age, gender, ethnicity, marital status, employment status, educational level, financial status
Rogero-Garcia & Rosenberg (2011)	Spain	The goals of this article are: to estimate the proportion of co-resident informal caregivers with paid (by the family) and unpaid (informal) support furnished by persons from outside the home; to identify the factors associated with both types of support and to quantify such support in terms of hours of help received over the preceding 4 weeks.	All households were initially identified with disabled persons aged 65 years or over, and then proceeded to select persons living in the same household who reported undertaking care activities for at least 10 min during the day to which the survey refers. Second, in order more easily to identify the person who was receiving help from the caregiver, only households having one disabled person aged 65 years or over were selected.	Informal caregivers *n*=404	Cross-tabulations, Chi-square, binomial logistic regression models	Spanish Time Use Survey, conducted by the National Institute of Statistics (2002–2003)	Age, gender, educational level, employment status, marital status, relationship, place of residence, support of others, household situation
Schulz et al. (2011)	Unites States of America	The purpose of this article is to address three important questions about choice among informal caregivers: to what extent do caregivers report having choice in taking on this role; what factors are associated with the perceived lack of choice; and how does this perception affect caregiver outcomes?	A random digit dial (RDD) sample stratified by geography to generate a set of telephone numbers proportionate to the population was used for those 1000 interviews. The design also called for oversamples of ethnic minority caregivers.	Informal caregivers *n*=1397	Bivariate x-square test, multivariate logistic regression, regression analysis, ordinary least square regression, binary logistic regression	National Alliance of Caregiving and the American Association of Retired Persons. Telephone survey	Age, gender, ethnicity, educational level, employment status, relationship, burden
Chang et al. (2016)	Singapore	This study aims to examine socio-demographic correlates of caregiving reactions and the associations of these experiences with caregiver psychological distress.	To be included in the study, the caregiver had to be a Singapore Citizen or Permanent Resident, aged 21 years and above, and able to read and comprehend English, Chinese, Malay or Tamil. Participants who spoke only dialects were excluded from the study.	Informal caregivers *n*=344	Linear regression, multivariable linear regression, Spearman’s rank correlation	Questionnaire through convenience sampling	Age, gender, ethnicity, employment status, relationship, financial status, self-rated health, educational level
Vincent-Onabajo, Ali & Hamzat (2013)	Nigeria	The aim of this study was to explore the quality of life of caregivers of community-dwelling stroke survivors in north-eastern Nigeria.	A caregiver was included in the study if he or she had the informal responsibility of looking after a stroke survivor in the home; gave verbal/written informed consent and understood written and verbal information in English language due to the unavailability of translated and validated versions of the main measure utilised in the study, namely the World Health Organization Quality of Life brief version questionnaire (WHOQoLBREF), in the two predominant indigenous languages (Hausa and Kanuri language) of the population in the study location.	Informal caregivers *n*=59	Kruskal–Wallis test	Cross-sectional questionnaire: World Health Organization Quality of Life brief version questionnaire (WHOQoLBREF)	Age, gender, educational level, relationship, employment status, self-rated health
Koker de (2009)	Belgium	The aim of this study is to add to the literature on the ‘supply side’ of informal care, by extending knowledge on the sociodemographic determinants of caregiving for a person living in the same household and caregiving for a person living in another household.	When studying the intensity of caregiving, informal carers with missing information on the frequency of at least one care task, are furthermore left out (N = 77). On the whole, respondents excluded from the analyses are more often female, older, lower educated, less frequently involved in paid work and in less good health. They are also less often living with children and more frequently living with a person who is not their partner or child, than respondents who are retained in the analyses.	Informal caregivers *n*=2559	Cross-tabulations, chi-square, multinomial logistic regression	Survey Care in Flanders, 2003	Age, gender, marital status, educational level, employment level, self-rated health, household situation
Weinland (2009)	United States of America	The aim of this study is to describe the lived experience of informal African American men providing care for a relative within the home and explores their definition of caregiver distress.	Not mentioned	African American male caregivers *n*=10	Thematic coding/analysis	In-depth personal interview	Age, gender, ethnicity, marital status, educational level, employment status, financial status, religiosity, relationship, self-rated health
Kenny, King, & Hall (2014)	Australia	The purpose was to investigate changes in health status after the commencement of care-giving relative to the change for similar non-carers over the same period, examining the effects of quantity, duration and other aspects of the care-giving context, which may exacerbate or moderate care-giving impacts.	Carers were excluded: if caregiving at Wave 1, if they had less than two consecutive caregiving waves or if the only pre-caregiving data were more than 2 years before care-giving; as there was no information on the duration of care-giving between annual observations, we restricted the study to carers with two or more consecutive care-giving observations on the assumption that the second annual care-giving observation represented a minimum care-giving period of more than 1 year.	Informal caregivers *n*=424	Logistic regression model	Household Income and Labour Dynamics in Australia	Age, gender, ethnicity, marital status, financial status, educational lever, employment status, household situation, self-rated health.
Vecchio (2008)	Australia	The aim of this study is to examine the use of respite services among carers of non-institutionalised individuals aged 15 and over with either profound or severe disabilities.	Based on the SDAC, the analysis was confined to primary carers of non-institutionalised people aged 15 and over who possessed either a profound or severe disability.	Informal caregivers *n*=243.690	Binary logistic regression	Australian Survey of Disability, Ageing and Carers (2003)	Age, gender, ethnicity, culture, employment status, relationship, financial status, household situation, support of others, place of residence
Stacey et al. (2016)	Australia	The aim of this study was to demonstrate the prevalence and demographics of adult carers aged 15 and over in the state of South Australia over 20 years between 1994 and 2014.	Not mentioned	Informal caregivers *n*=1.504	Age-Period Cohort (APC) analysis	Health Omnibus Survey	Age, gender, ethnicity, marital status, employment status, financial status, educational level

### Contextual dimensions

Many of the included studies described contextual dimensions in introducing their studies, embedding their study in a specific context. Distinctions were made, in the included articles, between dimensions in the historical, cultural and societal/political context.

Three articles [27–29] take account of the historical context influence: for example, it is argued that informal care is ‘as old as time’, but that it was often taken for granted by traditional attitudes towards family responsibilities until the later 20th century [27]. Historical context is also taken into account when explaining specific cultural situations that influence caregiving situations, for example how the post-colonial culture, which came forward in the role of Christian beliefs and values, influences care attitudes in Belize. ‘These beliefs were represented in the caregivers volition to assume a caregiver role’ [28, p. 17]. Or for example, Doebler (2017) studied the influence of the ‘Northern Ireland conflict’, which resulted in increased mental health issues, on caregiver burden.

Eight articles discussed the influence of cultural context on caregiving situations [27, 30–36]. It is often acknowledged that women are more likely to provide informal care than men [e.g. 28–31], which can be explained by the locally prevailing cultural context. For example, del Río-Lozano et al. [34] described that in Spain, ‘care attitudes can be sexist with women being pressured to provide care to others’ (p. 1507). Other studies underlined strong filial obligation norms influencing caregiving situations [27, 35, 36]. For example, ‘the cultural structure in many African countries encourages community and family care rather than institutional and nursing home care’ [35, p. 978]. Wang et al. (2016) [33] described that the unique endowment culture of the Uyghur and Kazakh, encompassing ‘honoring and respecting elders, helping and loving mutually and being filial towards parents’ (p. 19), results in family members taking care of disabled elderly in their own homes.

The description of the importance of family members caring for ill or aged relatives in Stacey et al. (2016) [27] is an example of the societal/political contexts’ influence on caregiver experiences. The authors described that now the importance of caring family members is being recognised in social policies in western countries, which leads to acknowledgement of informal care’s significant economic contribution. With this political shift, Stacey et al. conclude that caregivers in current societies are ‘recognized as a separate group in their own right’ [27, p. 15]. The influence of the societal/political context on informal care situations is mentioned in 21 articles. In general this is limited to ageing; an ageing population leads to increasing age-related heath issues, which consequently lead to higher and policy changes related to deinstitutionalisation [28, 30, 31, 35–45]. Some articles described the change in family structures caused by ageing, women’s growing labour force participation, and changing gender roles [28, 31, 32, 34, 37, 40, 41].

Several other contextual dimensions that influence caregiving situations were described, such as the influence of rurality [29, 34], migration [28], and welfare state regimes [33, 39, 46]. For example, Rogero-García and Rosenberg (2011) elaborate on the familyist welfare regime in Spain which is ‘characterized by the intense participation of the family in older people care responsibilities’ [46, p. 95]. Wang et al. (2016) explain that the lack of a strong welfare system in far western China enforces the endowment model within informal care ‘in which the family member personally cares for disabled elders in their homes’ [33].

Within these different context descriptions some attention is given to power relations and social inequality regarding caregiver experiences [33, 34, 45]. For example Verbakel et al. [45] describe that in policies, an increased reliance on informal care is assumed but simultaneously informal care remains hidden. This is ‘unfortunate because informal care responsibilities disproportionally fall on certain social groups, such as middle-aged women’ (p. 90). Another example comes from a cultural context where, ‘filial piety is the most distinct characteristic in the traditional old pension culture. Parents have the supreme power in the family’ [30, p. 18]. A lack of choice whether or not to become a caregiver was then associated with an increased risk of depression [33].

### Types of research questions

Three types of research question could be distinguished in the included articles. The first type focused on the profile of caregivers. In 11 studies, diversity was used to get a better understanding of the caregivers’ backgrounds [27, 28, 32, 34, 37, 41, 42, 45–48]; for example, to get a better profile of the group of caregivers providing co-resident care [28, 37] or of caregivers providing care to someone suffering from dementia [41].

The second type of research question focuses on the consequences of providing informal care [29–31, 33–36, 39, 40, 43, 44, 49–53]. Sixteen articles used diversity to understand better caregiving burden, caregiving health impact and/or quality of life of caregivers. Sometimes, a study focused on a specific group of caregivers and their burdens; for example, caregivers of persons suffering from schizophrenia [49] or caregivers in the palliative care context [52]. The last type of research question, studied in five articles, focused on caregiver coping [34, 38, 40, 50, 54]. Examples are differences in support needs, coping strategies, sources of satisfaction [50] and use of respite care [38].

Power structures and social inequity within informal care were not mentioned explicitly in the research questions. The research questions were mainly focused on the outcome and not framed from a contextualised and intersectional perspective. To formulate an intersectional informed research question it is important to consider which categories are relevant to include [16], and to address that intersections between these categories are open empirical questions [24].

### Used methodologies

The intersectional lens was used to analyse the used methodologies by looking at the sample of the studies, the method of data collection and the analytical approach, steps 3,4 and 6 from the coding scheme shown in Table I. In 25 studies, quantitative methods were used. Most of these studies used existing datasets in order to answer their research question. Sample sizes varied a lot, five studies were based on large datasets containing up to 378,365 respondents, for example by linking several national datasets [29, 30] or using European data [45, 49]. In these large datasets, often a subset was used containing information about caregivers. Besides the information about this group, information about other members of care networks or about non-caregivers was used as well. This shows the challenge described by Rouhani (2014) that the datasets were not designed a priori with intersectionality in mind and an analysis of intersections was only possible post data collection [16, p. 8].

Regarding the analysis, an additive approach was adopted in 15 of the quantitative studies. This type of analytical strategy enables researchers to quantify differences in social positioning within an identity group [2]. Using bivariate [27, 30, 35, 38, 41, 42, 46, 49, 54] or multivariate regression models [39, 40, 43, 45, 48, 52], the association between several independent variables and the outcome variable was investigated. A multiplicative approach was adopted in 11 studies [29, 32, 33, 36, 37, 44, 45, 47, 48, 50, 51]. In most of these studies, interaction terms were added to the multivariate linear or logistic regression analyses. Using this multiplicative approach allowed the researchers to analyse social and health inequities based on intersections of axis of diversity [16].

Although qualitative research is more compatible with intersectionality [22], as it allows for a greater understanding of people’s live experiences of complex inequities [16, p. 8], only three studies used qualitative methods. When qualitative methods were applied thematic analysis [28, 31] or sociological discourse analysis [34] was used. This way researchers aimed to preserve the uniqueness of caregivers’ experiences [31]. The studies mainly focused on describing the lived experiences of caregivers and explore and interpret the influence of the axis of different dimensions of diversity on, for example, caregiver distress [31], coping strategies, the choice to become a caregiver [34] or the perceived caregiver role [28].

### Dimensions of diversity

**Figure 2.**
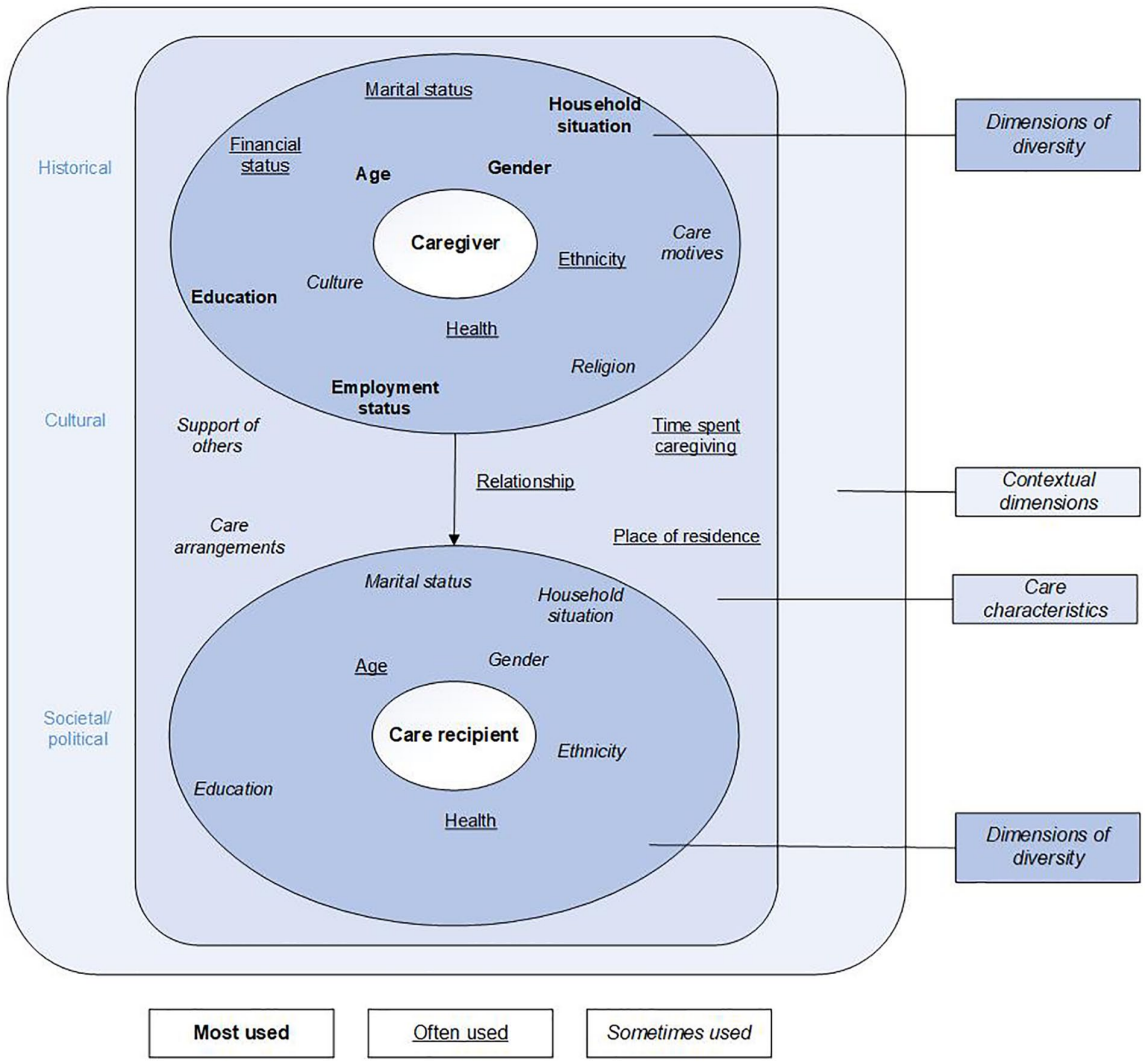
Visualisation of used dimensions of diversity in context.

When diversity was taken into account in informal care research, different dimensions of diversity were included as well as care characteristics. The dimensions of diversity of both the caregiver and the care recipient, care characteristics and the earlier mentioned contextual dimensions are visualised in Figure 2. Although intersectionality assumes contextualised interconnectedness of axes of diversity and does not view dimensions of diversity as single issues [16, 22, 55], this section first provides a description of the different dimensions of diversity which emerged from the included studies. As intersectionality demands in-depth reasoning about the study sample to enable researchers to think beyond existing categories [22], a description of categories studied may create space for the complexity of informal care [56].

As seen in Figure 2, a distinction was made between the caregivers’ and care recipients’ dimensions of diversity, which influence each other. In the description below *N* is used to indicate the number of included articles using the described dimensions of diversity. For caregivers, dimensions of diversity that were most often used were: age (*N*=28), gender (*N*=26), household situation, which means living with or without the care recipient (*N*=26), employment status (*N*=25), education level (*N*=24), financial and marital status (both *N*=18) and ethnicity, that is the use of race or country of birth as the determinant (*N*=15). Finally, a few studies also included religion, culture and care motives in their analysis.

For example, Wang and colleagues [33] investigated the prevalence and related factors of depression among low-income female Uyghur and Kazakh caregivers of disabled elders residing in China’s far west. Their study showed that demographic characteristics of caregivers, caregiver burden, and the care recipient’s impairment significantly correlated with depressive emotion among women caregivers. Chang and colleagues [36] used these characteristics to investigate the impact on caregiving reactions and the associations of these experiences with caregiver psychological distress; their study results showed differences between groups [36].

Regarding care recipients, the following dimensions of diversity can be distinguished: the impairment the care recipient has, the age or gender of the care recipient and other characteristics such as education level, marital status, ethnicity and household situation. Type of impairment of the care recipients was included the most (*N*=16). For example, authors used the type of disability (physical, psychological or both) [34], investigated whether care recipients’ impairment was experienced as mild or severe [38], the degree of cognitive decline and the type of dementia care recipients suffered from [41], and the influence on the caregiving situation. For example, Hastrup et al. investigated the correlation between the care recipients’ impairment and burden among caregivers, showing that caregivers who provide care to someone with a mental illness feel more burdened than others [43]. In addition, studies also included the care recipients’ age (*N*=10) and gender (*N*=7) while only a few studies also included the other dimensions.

Besides dimensions of diversity several care characteristics proved to be connected to the caregiving experience. The most commonly used care characteristic consists of relationship elements between the caregiver and the care recipient (*N*=16). Also, the time spent providing informal care (*N*=14), caregiver place of residence (e.g. living with or without the care recipient or rural or urban geography) (*N*=12), care arrangements (e.g. type of provided help) (*N*=9) and availability of support of others (*N*=7) were used to investigate differences in informal care situations.

### Intersections of dimensions of diversity

The intersectionality lens was used to see if intersections of diversity dimensions emerged and if so, how this was reflected. In 15 articles the included dimensions of diversity were studied as single issues. For example Vecchio [38] found that several factors were associated with not using respite care, for example being a wife or being younger in age [36]. Or, single issues were used to provide insight into the caregiving demographic of a particular region.

Thirteen articles studied the intersections of diversity dimensions and their impact on caregiver health, the caregiver experience or on the time spent caregiving. Seven articles used two or three dimensions of diversity [30, 32, 37, 39, 45, 47, 48]. In six articles, intersections between four or more dimensions of diversity were part of the analysis [28, 29, 31, 33, 34, 51]. Table III shows per article which intersections of diversity dimensions were analysed.

**Table III.** Representation of intersections of dimensions of diversity.

Study	Gender	Ethnicity	Caregiver	Time spent cergaguiving	Age	Employment status	Religion	Financial status	Education	Health care recipient	Relationship	Marital status	Place of resicdence	Age care recipient	Care arrangements	Geography	Supoort of others	Household situation
Wang et al. (2016)	x	x			x		x	x										
	x	x		x			x	x										
	x	x					x	x		x								
	x	x					x	x			x							
	x	x					x	x									x	
Vroman & Morency (2011)		x						x						x				x
		x		x						x				x				
		x					x							x				
Doebler, Ryan, Shortall & Maguire (2017)	x			x	x	x												
			x	x		x												
			x			x												
			x												x			
				x	x													
	x			x														
Kenny, King & Hall (2014)	x		x	x		x												
			x	x		x												
				x	x	x												
			x	x														
			x			x												
			x		x													
	x		x	x														
	x		x															
Verbakel et al. (2017)			x	x														
	x		x															
	x								x									
	x					x												
Ratcliffe, Lester, Couzner & Crotty (2012)			x									x						
			x										x					
Robards, Vlachantoni, Evandrou & Falkingham (2015)	x				x													
Tokunaga & Hashimoto (2017)	x				x													
	x											x		x				
	x					x												
Schmidt et al. (2016)						x			x		x							
Schulz et al. (2011)				x						x								
del Río-Lozano et al. (2013)	x					x									x			
	x		x															
	x				x				x							x		
	x				x				x							x		
	x				x				x							x		
	x				x													
	x									x	x							
Koker de (2009)					x								x					
											x		x					
												x	x					
Weinland (2009)	x	x					x					x						
	x	x					x				x							
	x	x					x										x	
	x	x					x	x										
	x	x					x								x			
	x	x					x											
**Total**	**30**	**14**	**14**	**14**	**12**	**11**	**7**	**7**	**6**	**5**	**5**	**4**	**4**	**3**	**3**	**3**	**2**	**1**

In general, gender is the most common single dimension of diversity used in an intersection (*N*=30) [29–34, 45, 51]. Regarding intersections, gender, ethnicity and religion and their relation to caregivers’ experience were the most studied (*N*=6). For example, Weinland [31] studied the lived experience of informal African American male caregivers. ‘Participants discussed their use of prayer, faith in God or religion as a coping mechanism. . . their spiritual beliefs guide African American male caregivers as they cope with the illness of their loved one’ [31, p. 20]. Sometimes other dimensions of diversity were added to this intersection, such as marital status, relationship, support of others, or care arrangements.

Second, gender, ethnicity and class or financial status were studied (*N*=5) in relation to the caregivers’ health. For example, Wang et al. (2016) [33] studied depression among low-income female Muslim Uyghur and Kazakh informal caregivers of disabled elders in far western China. ‘The results of this study indicated that the depressive emotion of female informal caregivers of disabled elders was primarily associated with the caregivers’ demographic characteristics. . . caregivers’ age and self-evaluation of health were each associated with an increased risk of depressive mood’ [33, p. 13]. Sometimes other dimensions of diversity were added to this intersection, such as age, marital status, relationship, support of others, or care arrangements. As Table III shows, several other patterns could be discovered while analysing which intersections of diversity dimensions were used in the included studies. For example, intersections of gender, age and level of education; gender and employment status; and employment status, being a caregiver and time spent caregiving were used.

Within the 13 studies that included intersections or dimensions of diversity, two articles made reference to power and social inequality [32, 34], both in relation to caregivers’ choice. Tokunaga and Hashimoto [32] described that ‘being female, low educational attainment, and being single are known to be associated with a lack of power in the household’ (p. 50). This influences the caregiving experience as this may lead to the fact that ‘informal caregiving is distributed in a biased way to women with less power in the household system’ (p. 52). According to del Río-Lozano et al. [34]: ‘women of a lower educational level living in rural areas expressed that they had no choice but to take on socially imposed caregiver roles’ (p. 1511). Both quotes clearly show how inequality plays a role in caregivers’ choice of becoming a caregiver based on different axis of dimensions of diversity, mostly based on gender and education level.

## Discussion

This scoping review of 28 articles explored how intersectionality is currently used within informal care research, and what such a perspective might add to informal care research. Within the included studies none of the articles explicitly mentioned an intersectionality perspective. However, two approaches have been used to study diversity in relation to caregiving experiences. First, diversity studied as a single issue, in which the influence of different dimensions of diversity on, for example, caregiver burden was examined separately and not in interaction with each other. Second, dimensions of diversity were (partially) studied in intersections in 13 of the included studies, but not explicitly framed from an intersectionality perspective.

In the included articles several aspects emerged that could be associated with intersectionality. For instance, most included quantitative studies used existing datasets, which often do not allow for the consideration of intersecting dimensions of diversity prior to data collection or for taking into account different categories with a reflection on the complexity of social life [22]. As a consequence, data on particular groups are missing, and the sample size may not be sufficient enough to fill all the cells in the analyses [16, 24]. ‘When developing an intersectionality-informed research question, researchers must consider which categories will be included’ [16, p. 5]. When an intersectional perspective is used statistical analysis can investigate whether ‘statistical interactions between inequity variables manifest significant effects above and beyond their main effects seen in the additive models’ [16, p. 11]. As another example, qualitative studies aimed to preserve the uniqueness of caregivers’ experiences [31] by analysing intersections of dimensions of diversity of caregiver experiences in relation to, for example, the outcome stress, burden or coping. Although this resulted in a rich description of caregiver experiences, an intersectional perspective could have brought a deeper level of analysis by paying attention to social inequities and power relations [22, 24].

Consequently, two aspects come forward which can be associated with intersectionality; namely, the description of contextual factors and a relation to power and social inequality. Intersectionality is grounded in from the assumption that research is context bound [20], and many included studies embedded their studies in their specific context [27–29, 35, 36]. However, most studies use dimensions of diversity rather as descriptives without analysing their meaning. Some account was taken of a deeper layer in which those categories harbour power relations that in and of itself shape the caregivers’ experience, for instance, when articles reference power or social inequality in relation to caregiver experiences [32–34, 45]. This was mainly done to put a study in a specific societal/political or cultural context. Del Río-Lozano et al. [34] suggest that ‘there are significant gender inequalities in caregiving driven by stereotypes and gender norms’ (p. 1515). Power relations are also mentioned in caregiver experiences in relation to different dimensions of diversity directly, providing a detailed and nuanced understanding of the lack of choice whether to become a caregiver and the influence of this lack of choice on caregiver health [32, 34].

Aspects of diversity in informal care in particular across their intersections and from a critical perspective are largely understudied. However, the analysis of the 28 included articles, from an intersectional perspective, showed several aspects that can further our understanding of caregiving experiences: caregiving experiences are unique, there are many different caregiver experiences, and that dimensions of diversity play a role [8, 14]. Gender was most often used in intersection with other dimensions of diversity. From a historical perspective informal care is gender-biased but obviously the group of caregivers is more diverse [6]. For example, when researching partner caregivers, Stacey et al. [27] showed that caregivers were predominantly women but looking at the intersection of gender, age and life course revealed that ‘from the age of 75 there were slightly more male primary caregivers’ and ‘after the age of 85, carers were frequently males caring for a disabled wife’ [27, p. 9]. It is evident that it becomes more and more important to pay attention to differences in the roles, expectations and needs of caregivers [4] in order to improve collaboration between caregivers and professionals and developing serviceable policies [8, 9].

Adopting an intersectional perspective can also clarify differences in caregiving experiences within and between groups [57]. For example, Verbakel et al. [45] describe the following intersection regarding informal care, ‘the demand for informal care is experienced mainly by middle-aged women’ (p. 94). When looking at women caregiver experiences from an intersectional perspective Giesbrecht et al. [6] explain that ‘by adopting an intersectional approach, it became apparent that women are not one homogenous group, but are complex and diverse individuals who simultaneously inhabit other distinct socioeconomic, cultural, political, and historical locations, and as such, their caregiving experiences are likely to vary dramatically’ [6, p. 2]. Their study provides deep insight into the structural positions of caregivers within Canadian society.

Using an intersectional perspective provides the opportunity for the intentional use of diversity dimensions from a relational and critical perspective, allowing researchers to dig deeper for whom and under what conditions the knowledge is ‘true’ or ‘valid’. For example, who is the most burdened or about whom is the least knowledge created. By doing so, power is made visible and diversity is translated as questioning inequality and structural disadvantages [24]. For example, Holmgren et al. argued that caregivers have to deal with different obligations, interests and power structures, which can create a feeling of being caught in between different expectations and structures [18]. ‘Becoming a caregiver relative means establishing oneself in a betweenship of a traditional gender power structure and a division of labor conditioning what male and female relatives “naturally” should be involved in, as well as how to perform care activities’ [18, p. 234]. When focus lies on mapping how people are made vulnerable, not necessarily being vulnerable, a representation of their voice and assessment of their situation from their perspective is required.

This scoping review has several limitations: first, only articles published in English or Dutch were included, therefore some studies may have been excluded. Second, several studies [6, 17–19, 58] that explicitly used intersectionality were not included in our review. Neither did they appear in the used databases with the used search strategy, nor in the reference lists of the included articles, or they did not meet our third inclusion criterion of using four or more dimensions of diversity. As these articles do provide valuable insights, they are used extensively in the introduction and the discussion. Several decisions were made in order to improve the strength of our study. First, a well-established method was used for reviewing, data extraction, summary and thematic synthesis [26]. Researcher triangulation was used in all phases of our study. Second, creating a thematic synthesis goes beyond the preliminary results and results in a higher level of evidence and understanding about the meaning of diversity in caregiving experiences [59]. Third, studies were gathered from four different electronic databases and represented different countries all looking at least at four different dimensions of diversity with regard to informal care. The search strategy, using four databases and a combination of MeSH and free-text terms, produced a heterogeneous set of studies.

Based on this scoping review several recommendations can be made for the future. First, the use of intersections of dimensions of diversity to interpret caregiver experiences must be amplified by encouraging researchers to incorporate an intersectionality perspective in a structured manner and integral to all parts of design. This way research can ‘develop more contextualized and reflexive understandings’ [24, p. 17] of caregiving experiences and effectively ‘capture social and health inequities’ [24, p. 15] in caregivers’ experiences. Second, this review focused on the caregivers’ perspective. Analysing the included studies showed that several authors also included care recipients’ dimensions of diversity. Based on the concept of positionality, which describes how identity influences and potentially biases understandings of and outlook on the world [60], it can be assumed that the dimensions of diversity of formal care providers also influence their perspective on informal care and their way of collaborating and supporting caregivers. Therefore, it would be interesting to include the perspective of formal care providers in further research. At the policy level, using an intersectionality lens can provide insight into the usual assumptions stakeholders make about the provision of care and to rethink collaborative care networks [18]. Taking diversity into account can support informal and formal care providers to understand each other better and thus improve collaboration and policy makers to create socially relevant, inclusive and effective policy solutions that contribute to social justice [16].

## Conclusions

Aspects of diversity are largely understudied in informal care research, in particular across their intersections and from a critical perspective. This review shows that studying dimensions of diversity across intersections makes room for a more nuanced understanding of informal care. However, adopting an intersectional perspective can clarify differences in and between informal care groups, providing the opportunity for the intentional use of diversity dimensions from a relational and critical perspective, making sure that not only different categories or social identities of caregivers are included in future studies, but the mutual relationships between these categories embedded in their specific context are actually studied, enabling a focus on power relations and social inequalities in informal care research. This provides insight into how caregivers are being made vulnerable, not necessarily how they are vulnerable. A representation of the voice of different caregivers within future caregiver research is required and an emphasis on health disparities in and between informal care groups, and let future informal care research be responsible for embedding the role that socio-structural dynamics play in informal care.
